# Latent tuberculosis testing through the ages: the search for a sleeping killer

**DOI:** 10.1152/ajplung.00217.2021

**Published:** 2022-02-16

**Authors:** Bri Kestler, Shannon K. Tyler

**Affiliations:** ^1^Department of Physiology and Cell Biology, College of Medicine, University of South Alabama, Mobile, Alabama; ^2^Department of Internal Medicine, Division of Infectious Diseases, College of Medicine, University of South Alabama, Mobile, Alabama

**Keywords:** IGRAs, Koch, PPD, tuberculin, tuberculosis

## Abstract

Tuberculosis has been present in the world’s population for as long as there has been written language. It is a disease known to the ancient Egyptians, Greeks, Romans, and Hebrews, but its etiology eluded the world for thousands of years. Even after the germ theory was accepted and early scientists hypothesized a pathogen as the cause, the identity of the sleeping killer in society remained a mystery. That is until Robert Koch was able to grow and visualize *Mycobacterium tuberculosis*. Koch introduced his Old Tuberculin solution as a diagnostic therapy of tuberculosis (TB), with the intent to reduce the number of infected persons and stop its spread. Old Tuberculin’s ability to treat TB proved minimal, but its diagnostic potential paved the way for more effective tests from von Pirquet, Calmette, Wolff-Eisner, and Mantoux. Florence Seibert set out to identify and purify the active principle in Koch’s Old Tuberculin and ended up creating purified protein derivative (PPD) tuberculin which is still used as the standard for the tuberculin skin test (TST). Interferon-γ release assays (IGRAs) are a more modern diagnostic tool for detecting latent TB infection that offer some benefits (and some disadvantages) to TST. TSTs and IGRAs can determine if an individual has been infected with *M. tuberculosis* but are equally unable to predict progression to active tuberculosis, the diagnosis of which relies on assessment of clinical symptoms, radiographic imaging, and sample culture.

Responsible for more deaths than any other infectious disease, tuberculosis (TB) has plagued mankind for millennia with ancient origins estimated around 150 million years ago (1, 2). Despite tremendous advances in diagnosis and treatment, TB remains a significant cause of death globally. According to the World Health Organization, TB killed 1.5 million people in 2020 (3). It is estimated that somewhere between one-quarter and one-third of the Earth’s human population is infected with *Mycobacterium tuberculosis* and more than 10 million new cases are reported every year (4–6). The majority of persons infected never develop the disease, but those who do risk becoming infectious to others, often before they are aware of their diagnosis. The current tenet places infected individuals into a binary categorization: latent TB infection (LTBI) and active TB (aTB). Those with LTBI are currently diagnosed by an immune response to tuberculous antigens without any evidence of clinical disease. They are asymptomatic and considered not infectious, but they have the risk of developing active disease. In them, this killer pathogen lies dormant, a hostage to the host’s immune defenses. An individual with LTBI has a roughly 10% risk of developing aTB over their lifetime, over half of which is in the first two years following infection. Accurate identification and treatment of LTBI is crucial to reducing the incidence of TB, as this prevents individuals from developing active tuberculosis (aTB) and becoming contagious. There are effective treatment regimens for LTBI which vary between 3- and 9-mo duration. Treatment of LTBI is of particular importance in persons who have increased risk for progression from LTBI to aTB, including immunocompromised persons and children. Though significant advances have been made, the ability to diagnose LTBI, an asymptomatic condition, remains problematic. We would like to review the history of latent TB testing, and thereby explore modern medicine’s search for a sleeping killer.

Archaeological evidence confirms human disease due to tuberculosis for at least 9,000 years. Egyptian art illustrates skeletal deformities consistent with TB, whereas written and archeological accounts of TB can be found in the early history of African, Asian, European, and American cultures (7, 8). The disease was called “phthisis” by the ancient Greeks, “tabes” by the ancient Romans, and “schachepheth” by the ancient Hebrews (with clear descriptions in the books of Deuteronomy and Leviticus). By the early 18th century, TB was hypothesized to be infectious in nature. Forty-six years after Antonie van Leeuwenhoek first described microscopic living organisms (53), or “animalcules,” Benjamin Marten, an English physician, provided an early theoretical statement on germ theory in 1720 by describing “animalcula or wonderfully minute living creatures'” as the cause (9). In 1865, Jean-Antoine Villemin (54), a French physician, hypothesized an infectious nature after observing TB rates to be higher in soldiers stationed in the barracks as opposed to those in the field, and noting that urban areas had a higher rate of infection than primitive landscapes (7). Villemin went on to conduct studies with rabbits inoculated with purulent material from a tuberculous cavity removed from a patient upon autopsy; 3 mo later, the rabbits demonstrated extensive TB on autopsy (7, 8). It was in the early 19th century, when the concept of latency first appeared; French and German physicians discovered pathologic changes of TB on autopsy in the lungs of persons who had no symptoms of TB at the time of death. René Laennec, the French physician who invented the stethoscope, reported tubercles in patients “in whom the phthisis had always been latent” in the first edition of his seminal contribution “De l’auscultation mediate” in 1819 (10). His further work inferred that the majority of those with phthisis were without symptoms. Until this time, TB had been considered uniformly fatal.

In 1882, Robert Koch, a German physician, using a weak solution of methylene blue provided by Paul Ehrlich and Weigert’s methods of staining with Bismarck brown, was able to visualize and isolate *M. tuberculosis*. He went onto culture the bacillus in ox and sheep sera and reproduced the disease in laboratory animals (7, 8). The next years saw Koch focus on creating a cure for TB, now that he had identified the culprit. In 1890, he claimed to have isolated a substance from the “tubercle bacilli”' that could cure patients with TB, and he called it tuberculin (8). Tuberculin was described as a “brownish transparent fluid” that, he proposed, protected guinea pigs against TB and also cured established disease (11, 12). Speculation and excitement rose in the months following his discovery and forced Koch to respond to questions regarding tuberculin’s efficacy earlier than he would have liked. In a November 1890 article, Koch discussed his original intention of completing his research and gaining experience on the administration of tuberculin, before publishing large-scale results (13), and additional communications penned in January 1891 described its curative effect and promise as a diagnostic aide (14).

Koch’s remedy for TB was created by growing tubercle bacilli in a medium of glycerinated beef broth and killing the organism with heat in a 100°C flowing steam cabinet. Koch would then concentrate the extract to one-tenth of its original volume (11, 12, 14). Koch was unsure of the composition of the “active principle” contained within the solution but believed it was a necrotizing agent. His hypothesized mode of action for tuberculin included adding “active principle” into the body via injection, where it would necrose tissues and disturb their growth, causing them to die off much sooner than under ordinary conditions (14). Koch acknowledged the remedy could be harmful in patients with extremely advanced cases, but believed deaths only occurred in “exceptional cases” (14).

On January 7, 1891, at a Berlin Medical Society meeting, Rudolf Virchow, a German physician and pathologist, shared autopsy findings of 21 patients who died shortly after receiving Koch’s remedy (15). The majority of the examined patients had phthisis (pulmonary TB), and instead of transient inflammation, these patients experienced intense inflammatory processes, hemorrhagic infiltrations, and active proliferations (15). Virchow noted extensive changes to the lungs and deduced if the tissue surrounding the bacilli is broken down, the bacilli could be mobilized, and “may give rise to new foci in other places” (15). He also commented that Koch’s remedy led to necrosis of intestinal ulcers and respiratory tubercles that loosened mass beyond the patient’s ability to expel. Virchow argued that in most of his autopsies he visualized “no evidence of retrogressive changes to the tubercles” (15), as was postulated by Koch.

## EARLY DIAGNOSTIC TESTS USING TUBERCULIN

In contrast to his statements about the therapeutic effect of tuberculin, Koch’s statements regarding use of tuberculin as a diagnostic tool held true, and the subcutaneous tuberculin test was administered by physicians, in hopes of identifying patients with asymptomatic tuberculosis. In Koch’s initial communication on his tuberculosis remedy, he observed that healthy patients given a 0.01 cm^3^ of tuberculin had a mild reaction, if at all. This reaction was much more severe in patients with tuberculosis, as they had a generalized reaction of fever, rigors, arthralgias, fatigue, coughing, and nausea and vomiting, which began a few hours after tuberculin injection. Patients with tuberculosis also had a local reaction at the injection site, with the skin becoming swollen, red, and painful (13).

During this time Viennese pediatrician Clemens von Pirquet was studying host reactions to foreign substances and coined the terms “allergy,” “allergen,” and “serum sickness.” He recognized that sensitization led to an accelerated response after subsequent exposure to an allergen and correlated this to the different effects of primary and secondary smallpox vaccines (16). Pirquet believed that the skin changes at the inoculation site of Koch’s tuberculin test was a localized reaction due to previous exposure to *M. tuberculosis* and hypothesized that a safer test could be created relying on this local reaction (8, 16). In 1907, von Pirquet publicized his tuberculin test that used two solutions of Koch’s Old Tuberculin, one diluted to 25% with a carbolic acid and normal saline diluting solution and the other of undiluted tuberculin. Three areas on the patient’s forearm were “scarified,” about two inches apart from one another. Of the three scarification sites, one had a few drops of the 25% diluted solution placed on it, the second site used the undiluted tuberculin solution, and the last site used the carbolic acid and normal saline diluting solution as a control (17, 18).

Two years later, von Pirquet published an extensive study on the patient’s reactions and his observations since the Pirquet’s tuberculin test was implemented by healthcare workers. He carefully identified the limitations of his test, noting that patients with measles were unable to respond to the tuberculin test, patients with miliary tuberculosis often tested negative, children less than two years of age had a doubtful or false-negative test, and skin test positivity regressed in the last week in half of fatal cases (16). Based on his detailed records, von Pirquet was able to distinguish the first limitations of a skin-based tuberculosis test. von Pirquet also coined the term “latent tuberculosis” by comparing a patient’s clinical, pathological, and radiological results to their skin reactions, recognizing that healthy children were also infected (16).

The same year von Pirquet published his test, Albert Calmette, a French physician, and Alfred Wolff-Eisner, a German physician, both independently suggested a conjunctival tuberculin reaction could be used as a diagnostic test for tuberculosis. Calmette first prepared a precipitation of the crude tuberculin to remove the glycerin and beef extract salts, as he believed they could be conjunctival irritants by themselves. The precipitate was dissolved in normal saline solution to create a 1% tuberculin solution. A drop of this solution was placed in a patient’s eye and a positive test would yield a conjunctival reaction consisting of erythema, eyelid edema, and discharge; a noninfected patient would not have a response (18–21).

Individual modifications to the von Pirquet test and ophthalmo-tuberculin test were implemented, as physicians believed that procedural changes could increase accuracy. Modifications included altering the concentration of the tuberculin solution, different methods for scarification, and varying time points the test was read for positivity (17–24). Physicians investigated these tests individually and also performed side by side comparison studies to determine their efficacy (17–22, 24, 25). These physician-to-physician differences impacted the value of the test by altering the amount of tuberculin absorbed by the patient, reading tests prematurely, and identifying a normal wound-healing response as a positive reaction. Altered testing procedures created difficulty when comparing the ability of these tests to recognize a true patient with tuberculosis, and many researchers ended up with varying positive reactions among similar patient populations. Researchers noted a moderate reaction in healthy adults with a 1% ophthalmic solution administered during the Calmette test. To counter this false-positive, most physicians began to use weaker solutions of 0.33% or 0.5% strength. The weakest solution would be placed in one eye and if the patient did not react, a drop of a stronger solution would be instilled in the other eye to confirm the negative test (18–21, 24). At first, the ophthalmo-tuberculin test was praised for its easy administration, consistent results within 24 h, and noninvasive nature, with the only contraindications being those of active eye conditions (18–21). Within the first year of the ophthalmo-tuberculin test in circulation, cases of conjunctivitis and keratitis began to occur after administration of the test. Physicians began to use the von Pirquet test more frequently, as it did not have any documented reports of harmful results, which did not hold true to the conjunctival test (18–21, 24).

## THE BEGINNING OF STANDARDIZATION

In 1908, Charles Mantoux, a French physician, developed an intradermal method of tuberculin testing, using a “sterilizable Pravaz syringe, with a graduated rod and fitted with a cursor, that is to say the current model, and to a fine needle” (26). The needle was advanced in a parallel fashion, to keep the solution more superficial, and 0.001 mg of a 1:5,000 solution, made with tuberculin stock and physiological water, was injected under the epidermis creating a wheal (26). Mantoux noted that the local reaction is at its peak after 48 h of injection and the “dimension of the infiltrated region, which are rarely smaller than a 50-cent piece, often exceed those of a 2-franc piece” (26). Mantoux’s method helped create a more consistent model for delivering a known amount of tuberculin solution and the most appropriate time to look for a positive reaction, but the criteria for a positive test was yet to be established.

The following decades saw researchers pitting von Pirquet’s dermal method and Mantoux’s intracutaneous methods against one another, to determine which test was superior. Results from these comparison studies demonstrated the Mantoux test to be more reliable with ∼80% of patients with tuberculous testing positive to the intradermal method, whereas only 50% of patients with tuberculosis tested positive with the von Pirquet method (27–29). During the early 1920s, physicians came to a general consensus on the criteria for a positive Mantoux test. Forty-eight hours after inoculation, the site was examined for induration and if present, the diameter of the indurated tissue was measured, with ≥ 5 mm indicating a positive test (28, 29).

## THE ANSWER IS IN THE PROTEIN

Florence Seibert was a biochemist who sought to identify and purify the “active principle” in Koch’s Old Tuberculin, hoping to develop a better solution for tuberculin skin tests (30). Tuberculin was still being manufactured using a glycerinated beef broth medium for culture of *M. tuberculosis*. Seibert verified that the bacilli would grow quite well on a synthetic medium (Long’s), which contained no protein and only solutes such as asparagine, ammonium citrate, glycerol, and inorganic salts (31). Seibert’s early studies investigated the filtrates of *M. tuberculosis* cultures, and with zonal electrophoresis and ultracentrifugation she was able to examine this topic more deeply (30). One of her first questions was whether the active principle was or was not a protein, and through a variety of techniques she was able to identify key attributes of the active principle (31). When protein was present in the culture medium, there was also tuberculin activity, but if the protein was removed by a thick dialysis membrane or degradation by trypsin or pepsin, the tuberculin activity was lost (31). Crystallization of the active principle would occur through the usual method for crystalizing egg albumin and the potency of tuberculin activity increased with subsequent recrystallizations. The active principle also behaved like a protein during sedimentation and electrophoresis, and protein molecules of different molecular sizes were active as well (31).

After it was determined that the active principle was a protein, Seibert set out to isolate it through precipitation studies using *M. tuberculosis* grown in Long’s medium. Acetic acid, ammonium sulfate, and trichloroacetic acid were used to precipitate the protein out, with ammonium sulfate and trichloroacetic acid being the most convenient and effective agents (11, 31, 32). Guinea pigs and rabbits were injected with tuberculin precipitants from ammonium sulfate and trichloroacetic acid, and both agents were found to be highly antigenic. Seibert was concerned that the increased antigenicity of these agents could result in false-positive reactions in humans, so she turned to Koch’s Old Tuberculin in attempts to purify a reagent with less antigenicity (31). After concentrating filtrates from cultures of Koch’s Old Tuberculin grown on synthetic medium, she removed the salts and glycerol with ultrafiltration. Removal of carbohydrates required further precipitation by trichloroacetic acid, and ether was used to remove acids and dry the compound. This product was found to be as pure as Seibert’s original precipitants with trichloroacetic acid, but less antigenic. Seibert called this product purified protein derivative (PPD) tuberculin (31, 32). This product worked well for skin testing; as it could be manufactured into a quantitative tablet, it was stable for many years in a dried form, and it proved to be a comparatively small molecule.

Two events occurred that caused Seibert to review her PPD production protocols. The National Tuberculosis Association placed a request with her laboratory for the preparation of a large quantity of PPD to be used as an official standard, and the potency of PPD made in other laboratories was found to be inferior (12, 30, 31). Seibert hypothesized that chemical denaturation was the cause of the weaker PPD in other laboratories, and her revised method focused on identifying steps in her previous protocol where denaturing could occur. She believed less denaturation would take place if the procedure was performed in a cold room, steam was not used for evaporation, a weaker acid aided precipitation, and drying did not use ether (31, 32). Electrophoretic studies of PPD validated that the compound contained more nucleic acid and other proteins than Seibert would have liked, and precipitation in an alkaline environment (>pH 5.0) helped separate these components (31, 32). The final procedure involved precipitation by half saturation of ammonium sulfate at a pH of 7.0 in 4–5°C temperatures, without steam evaporation, and final drying from a frozen state. Analysis and animal testing of the new PPD proved it to be of greater purity and potency; this new PPD could trigger the same reaction as old PPD at half the dose (31). The standard for all PPD preparations is Tuberculin PPD lot number 49608, or PPD-S (PPD-Standard) (11, 12, 33). Aliquots of this lot, prepared by Seibert in 1941, were sent to various governmental and commercial entities for use in research, and in 1952, PPD-S was adopted as the International standard for purified protein derivative of mammalian tuberculin by the World Health Organization (12). Seibert’s PPD allowed for the diagnosis of LTBI, an immunologic determination that an asymptomatic person has been infected with *M. tuberculosis*.

It was during this time that the first effective chemotherapeutic (antibiotic) treatment for aTB was developed, first with streptomycin and para-aminosalicyclic acid (PAS) in the mid-1940s, followed by isoniazid (INH) in 1952. Before this the treatment for aTB was primarily sanatorium care, which relied mostly on bedrest and fresh air. The first clinical trials using chemotherapeutic agents to treat LTBI began in 1956, using INH among 8,081 villagers in Greenland, followed by trials among Inuit and African populations, along with risk groups in the United States and Europe (34). These trials confirmed that treatment of LTBI prevented aTB among populations at high risk. Isoniazid continues to be a preferred treatment for LTBI worldwide to this day.

## SCIENTIFIC PREMISE AND ADMINISTRATION OF THE TUBERCULOSIS SKIN TEST

After inhalation of an air particle that contains tubercle bacilli by an immunocompetent person, an initial sensitization phase is initiated. Alveolar macrophages encounter the bacilli, and the bacilli enters the macrophage via endocytosis (35). *M. tuberculosis* uses many mechanisms to evade host response and can replicate inside macrophages early in the infection. An antigen presenting cell that has phagocytized *M. tuberculosis* will travel to a regional lymph node, process the antigen, and present the processed antigen fragments on an major histocompatibility complex II (MHC II) platform to naive helper T cells (36). This activates the naive helper T cell to differentiate into a Th1 cell and travel to the site of infection (37, 38). Th1 cells release interferon-gamma (IFN-γ) that activates macrophages, increasing macrophage phagocytic capability and interleukin-12 (IL-12) release. IL-12 facilitates clonal expansion in Th1 cells, leading to a large group of sensitized Th1 cells (39).

At this point, an immunocompetent patient should be able to contain an *M. tuberculosis* infection due to an adequate T cell immunity. However, if a patient’s T cell immunity becomes inadequate before a second exposure, either through the environment or a release from a once contained bacilli tissue depot, a more aggressive Th1 response will occur (37). Sensitized, antigen-experienced Th1 cells will now release more IFN-γ, increasing activation of macrophages (40). Activated macrophages release tumor necrosis factor alpha (TNF-α) which increases recruitment of monocytes to the site of infection and surrounding tissues (41). Increased activation of macrophages by IFN-γ results in increased TNF-α release, prolonged recruitment of monocytes, and production of inflammatory cytokines (39, 41). An excessive chemokine release and inflammatory setting causes activated tissue macrophages to take on an epithelioid state or fuse to form multinucleated giant cells. These cells, as well as sensitized Th1 cells, surround the antigen and form a granuloma with a caseous core.

When a patient presents for a tuberculin (Mantoux) skin test, 0.1 mL of five test unit (TU) strength PPD is injected intradermally into the patient’s forearm, to form a wheal. If the patient has previously been infected with tuberculosis, a delayed-type hypersensitivity (DTH) response begins. Second exposure of sensitized Th1 cells to intradermal PPD triggers release of interleukin-2 (IL-2) and causes Th1-cell clonal expansion, and release of IFN-γ to activate macrophages. The delayed nature of this response is based on the time required for recruitment of Th1 cells and activated macrophages to the site of the intradermal PPD, which takes between 24 and 48 h. Macrophages release proinflammatory cytokines such as TNF-α, interleukin-1 (IL-1), and interleukin-6 (IL-6) leading to erythema and induration of the inoculation site caused by vasodilation, vascular permeability, and infiltration by immune cells. The patient will return in 48–72 h to have their tuberculosis skin test (TST) read by a healthcare provider. The injection site is palpated for induration, and, if present, the widest portion of the induration is measured with a flexible ruler. Based on a patient’s comorbidities or TB exposure history, an induration measurement of 5–15 mm is considered positive. It takes 2–10 wk after initial TB exposure for a patient’s system to be sensitized to cause a positive PPD (12).

## LIMITATIONS OF THE TUBERCULOSIS SKIN TEST

There are multiple causes of a false-negative TST. An average of 10%–25% of patients with active TB do not react to the TST (12). Iatrogenic causes include improper storage or dilution of the PPD as well as poor TST administration and/or interpretation techniques. The variability in measuring induration among even experienced TST readers is 15% (12, 42). Patient factors such as extremes in age, immunocompromised state, concurrent infections, lymphoid disorders, or nutritional or metabolic derangements increase the likelihood of a negative test result. False-negative rates were found to be ≥50% among patients critically ill with disseminated TB (12, 42). Repeated testing of an uninfected person does not sensitize them to tuberculin, but sensitivity to tuberculin after infection may wane. This can cause a patient to have a negative TST if they were initially infected many years ago. A booster phenomenon occurs when the first PPD injection primes their weakly sensitized immune system to positively respond to a second TST administered 1–3 wk later. If this two-step TST is not performed, a patient may be erroneously classified as uninfected (12, 33).

The PPD used in TST has historically been refined and purified but still consists of a mixture of mycobacterial antigens, many of which are shared with proteins from nontuberculous environmental mycobacteria and the strain of *Mycobacterium bovis* used in the bacilli Calmette–Guérin (BCG) vaccine that is administered to persons living in regions with endemic TB. Cross sensitizations of the PPD mycobacterial antigens causes some nontuberculous mycobacteria, such as *Mycobacterium avian, Mycobacterium intracellulare*, *Mycobacterium scrofulaceum*, and *Mycobacterium kansasii* to elicit an erroneously positive TST result. The BCG vaccine is the only tuberculosis vaccine and uses a family of live attenuated strains of *Mycobacterium bovis* (43). The *M. bovis* BCG Ag85 antigen complex is present in all mycobacterial species and binds fibronectin to facilitate cell adhesion on macrophages (44). The intent of BCG vaccination is to decrease a patient’s risk of developing active TB by administering these mycobacterial antigens to trigger an immune response similar to that induced by *M. tuberculosis* infection (43, 44). The goal is to provide protective immunity against active infection by *M. tuberculosis* through the response to *M. bovis* antigens (12, 33, 42). The BCG vaccine acts as a sensitization step and subsequent exposure to mycobacterial antigens in the PPD can cause a local reaction that is read as positive but is incorrect, as the patient has been primed with *M. bovis* antigens and not infected with *M. tuberculosis*. False-positive TST due to BCG vaccination is most common in individuals who were vaccinated after infancy and those who received multiple (booster) inoculations (45). TST testing can still be accurately performed in those who only received the vaccine as infants. BCG has also been used for decades as adjuvant therapy for certain types of bladder cancer; over half of patients who have received this therapy will have a positive TST reading (46, 47).

The HIV/AIDS epidemic clearly demonstrated the importance of cellular immunity in preventing the progression from LTBI to aTB. Whereas the risk of someone with LTBI developing aTB is estimated at 5%–10% over the course of their lifetime, the risk for a person living with HIV is estimated at 5%–10% per year (1). Unfortunately, HIV infection is also associated with a higher likelihood of a false-negative TST, especially in those with more advanced disease. HIV led to a dramatic upsurge in global TB (most notably in sub-Saharan Africa), with cases peaking around 2005. Other comorbid conditions have been identified that significantly contribute to the risk of developing aTB, including diabetes mellitus, malnutrition and/or low body weight, smoking, chronic pulmonary disease, injection drug use, and the use of immunosuppressive medications (1, 2).

## INTRODUCTION OF SERUM TESTING

The TST has been the standard immunodiagnostic test to detect TB infection for many decades but due to its limitations, an *M. tuberculosis* antigen-specific IFN-γ release assay (IGRA) was developed in the 1990s. Currently, there are two major IGRAs available: the QuantiFERON-TB Gold In-Tube Plus (QFT-GIT Plus) assay and the T-SPOT.TB assay. The former is performed on whole blood and the latter on peripheral blood mononuclear cells (PBMCs). These tests measure immune response (type IV or delayed-type hypersensitivity) to specific mycobacterial protein antigens. The currently used IGRAs rely on antigens (i.e., early secretory antigenic target-6 kDa (ESAT-6), culture filtrate protein-10 kDa (CFP-10), and Rv2654c (TB7.7) antigen] located in regions not present in most nontuberculosis mycobacteria (NTM), rendering the IGRAs more specific than the TST (4). BCG vaccination or treatment does not cause a positive IGRA, but infection with some NTM that infect humans (*Mycobacterium marinum, Mycobacterium szulgai, Mycobacterium flavescens*, *M. kansasii*, and possibly *Mycobacterium leprae*) can cause a positive IGRA as those organisms share gene sequences that encode for ESAT-6 or CFP-10. For the IGRA to be performed, a blood sample is incubated with antigens and controls. It requires that cells remain viable and functional during the completion of the incubation period. Results are reported as positive, negative, or uninterpretable (indeterminate, borderline, or invalid depending on test manufacturer) and are usually available in 24–48 h. Specificity is estimated at >95% and sensitivity 80%–90%, and like TST, IGRA sensitivity is diminished in those who are immunocompromised.

## LIMITATIONS OF THE IGRAs

Although IGRAs are more specific than the TST, as they are not affected by BCG or most nontuberculous mycobacterium, they have other limitations. The reagent costs are significantly higher than TST, and the tests require phlebotomy, laboratory equipment, and technical expertise for collection, processing, and assay. Interpretation of serial IGRA tests is problematic as there is an unexpectedly high rate of conversions (negative to positive) and reversions (positive to negative). Many of these are centered around the manufacturer-recommended cut-point. As a result of poor reproducibility, most experts do not recommend serial IGRA testing, especially among low-risk populations. There is still fairly limited data on the use of IGRAs in very young children, immunocompromised persons, and those who were recently exposed to tuberculosis.

## LIMITATIONS OF LTBI TESTING AS A WHOLE

TSTs and IGRAs can determine whether individuals have been infected with *M. tuberculosis* but are equally unable to predict who will progress to active tuberculosis. A negative result from either test cannot rule out the possibility of infection with *M. tuberculosis.* The value of either test is dependent on pretest probability of infection, and screening for LTBI should be reserved for those who are at a sufficient risk for developing aTB: “intention to test is intention to treat” is the widely accepted mantra. As there is no gold standard for LTBI diagnosis, sensitivity and specificity cannot be accurately calculated. The currently accepted sensitivity calculations are based on individuals with culture-confirmed aTB and must thereby be interpreted with caution. One meta-analysis of studies showed a pooled sensitivity of 78% for QuantiFERON-TB Gold, 70% for QuantiFERON-TB Gold In-Tube, 90% for T-SPOT.TB, and 77% for TST (48). Other studies have shown higher sensitivity rates. Perhaps better-quality markers in LTBI testing are positive and negative predictive values. The current available tests are good at determining who is unlikely to develop aTB with high negative predictive values (99.7% for IGRA and 99.4% for TST) but poor at determining who will develop aTB with very low positive predictive values (2.7% for IGRA and 1.5% for TST) (48).

The choice of which LTBI test to be used is based on setting, availability, and cost. There are algorithms to help determine which test to use and online calculators that can help determine a patient’s overall risk for developing aTB (49, 50). Certain situations favor one test over the other. IGRAs are preferred in persons who have received BCG and those who have difficulty returning to a healthcare setting to have the test read. TST is preferred for children less than two years of age and for serial testing in populations exposed to TB. TST is also less expensive and requires less infrastructure to perform, making it more widely available in resource-limited settings. In general, dual testing (using both IGRA and TST) is not recommended; however, this practice is not uncommon if the initial test results are unclear or unexpected. Among individuals who are at very high risk of infection and progression to aTB, some recommend following a negative TST or IGRA with the other test (with treatment recommended if either test is positive) (51). In all patients who test positive for LTBI, whether by TST or IGRA, it is imperative that the possibility of aTB is ruled out by clinical assessment before the initiation of LTBI treatment to prevent the development of drug-resistant tuberculosis.

The diagnosis of aTB relies on assessment of clinical symptoms, radiographic testing, and laboratory testing (52). The gold standard for diagnosis remains culture, a process that can take weeks for the organism to grow. Significant advancements in testing have been made in recent years that allow for a more rapid diagnosis of aTB. Molecular testing of sputum allows for rapid detection *of M. tuberculosis* DNA as well as common mutations that allow for drug resistance. Urine-based antigen testing for lipoarabinomannan (urine LAM) can detect pulmonary and extra-pulmonary disease in HIV-positive individuals. These innovations have allowed for more rapid diagnosis and treatment for people with active TB.

## SUMMARY

The ability to detect latent infection has been fundamental in the global fight against mankind’s greatest infectious enemy. LTBI is unique among infections as it is diagnosed by the “inability” to detect the organism. The TST is still a widely used tool for the diagnosis of latent tuberculosis, even though it relies on methods created over a 100 years ago and a protein derivative purified 80 years ago. The development of interferon-γ release assays has been useful in overcoming some of the limitations of TST; but as a whole, diagnosis remains flawed, relying on tests that measure the immune response to the infection. Recently there has been growing interest in the use of novel *M. tuberculosis* stage-specific antigens for the diagnosis of LTBI and aTB, but there is limited evidence that these may improve sensitivity and lead to the ability to discriminate LTBI from aTB. Although it is clear that the development of aTB most commonly occurs within the first 2 years of infection and those certain conditions predispose individuals for reactivation, there is no test available that can determine who has cleared the infection and there is no test that can effectively rule out infection. Until better diagnostic tools are widely available, the sleeping killer remains elusive.

## DISCLOSURES

No conflicts of interest, financial or otherwise, are declared by the authors.

## AUTHOR CONTRIBUTIONS

B.K. drafted manuscript; B.K. and S.K.T. edited and revised manuscript; B.K. and S.K.T. approved final version of manuscript. 
